# The Number of Resected Lymph Nodes (nLNs) Combined with Tumor Size as a Prognostic Factor in Patients with Pathologic N0 and Nx Non-Small Cell Lung Cancer

**DOI:** 10.1371/journal.pone.0073220

**Published:** 2013-09-04

**Authors:** Miaomiao Yang, Hongxin Cao, Xi Guo, Tiehong Zhang, Pingping Hu, Jiajun Du, Qi Liu

**Affiliations:** 1 Institute of Oncology, Provincial Hospital Affiliated to Shandong University, Shandong University, Jinan, P.R. China; 2 Department of Medical Oncology, Yantai Yuhuangding Hospital, Qingdao University School of Medicine, Yantai, P.R. China; 3 Department of Intensive Care Medicine, Qilu Hospital, Shandong University, Jinan, P.R. China; University of Nebraska Medical Center, United States of America

## Abstract

**Background:**

The prognostic role of the number of resected lymph nodes (nLNs) in pathologic N0 (lymph node negative) and Nx (no lymph node examined) non-small cell lung cancer (NSCLC) patients remains uncertain. Guidelines for optimal nLNs have not been established. In the current study, we evaluated whether a higher number of resected lymph nodes (LNs) results in better survival in different tumor size categories among NSCLC patients without metastatic LNs.

**Method:**

A retrospective study was conducted. Based on nLNs (LN = 0, 1–7, >7) and tumor size (T_a_: ≤3.5cm, T_b_: >3.5cm) during surgery, patients were categorized into 6 groups (LN_0_T_a_, LN_0_T_b_, LN_1–7_T_a_, LN_1–7_T_b_, LN_7-_T_a_ and LN_7-_T_b_). Survival and multivariate analyses were carried out to determine whether nLNs combined with tumor size was significant for overall survival (OS) or disease free survival (DFS) after adjusting for potential confounders.

**Results:**

A total of 428 patients were enrolled in the study. Multivariate analysis demonstrated that nLNs, tumor size and pathological stage were the independent prognosticators for OS and DFS. Data from our study suggested that lung cancer lymphadenectomy with more than 7 LNs removed should be considered a benchmark for surgery or pathology at an early stage. Survival was significantly better in the LN_7-_T_a_ group, compared with other 5 groups (*p*<0.001).

**Conclusions:**

The combined predictor (nLNs combined with tumor size) is an independent prognostic factor and a reasonable stratification criterion in patients with pathologic N0 and Nx NSCLC. The validation of our finding is warranted in further investigation.

## Introduction

Lung cancer retains the status of leading cause of cancer-related deaths in both men and women in the United States, with 159,480 estimated deaths in 2013 [1]. Despite optimal treatment, the 5-year overall survival rate (approximately 16%) of lung cancer has shown the least improvement compared with other cancers. Currently, lymph node status is regarded as a valid risk stratification tool and the most powerful prognostic factor for patients with lung cancer [2]. However, 44% of patients with pathological node negative (pN0) disease still die within 5 years.

Indeed, the therapeutic effect of the extent of lymph node (LN) dissection and the optimal number of examined lymph nodes (nLNs) during surgery in patients with non-small cell lung cancer (NSCLC) remains controversial[3]–[6]. Examining more LNs may eliminate micrometastatic lymph nodes, increase the likelihood of accurate staging and then influence the survival data [7]. Patients who had no LNs examined, which termed ‘pathologic Nx’ (pNx), are often excluded to analyze the correlation between nLNs and outcomes. As these patients are often treated as pathologic N0 in clinical practice, they were comparative and have some commonalities on characteristics with pN0 patients.

Furthermore, tumor size has been directly related to cancer lethality and acted as a significant predictor of LN metastasis [8]. Within our dataset, both nLNs and tumor size have been taken into consideration in the NSCLC patients without metastatic lymph nodes. The goal of our study, therefore, was to evaluate the prognostic impact of nLNs combined with tumor size. To achieve the goal, we analyzed a series of 428 NSCLC patients who were surgically treated and identified as pN0 or pNx.

## Materials and Methods

### Patients

Clinical records and official pathological reports of the consecutive patients who underwent surgical resection for primary lung cancer at our institute between 2006 and 2009 were reviewed retrospectively. Surgery was performed by experienced surgeons in the same team.

**Figure 1:**
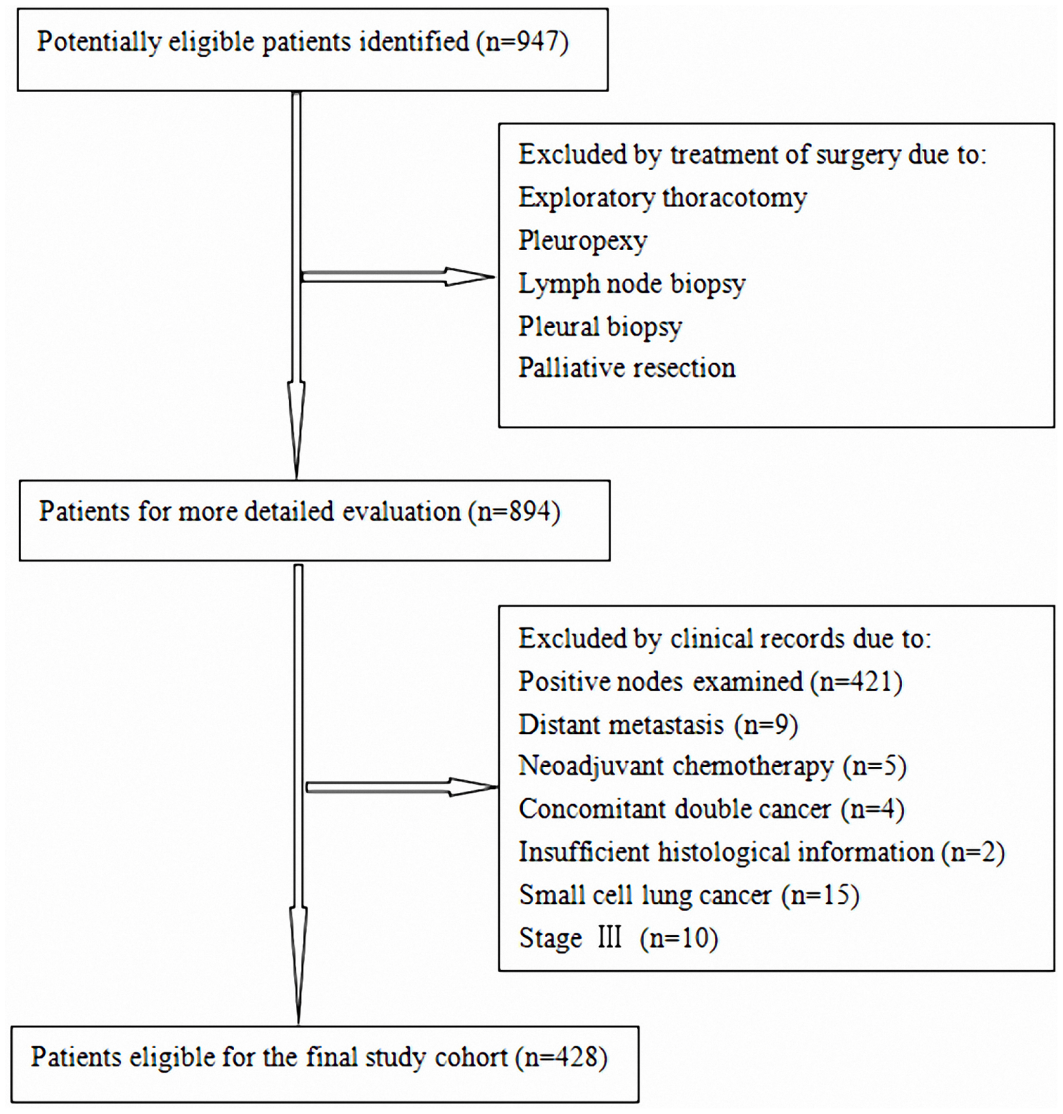
Flow chart of the cohort selection.

The inclusion criteria were as follows: therapy by surgery and absence of positive LN in pathologic specimen. Patients who had small cell lung cancer, distant metastasis, preoperative chemotherapy or radiotherapy, concomitant double cancer, surgical margin status positive, died within one month and those with insufficient histological information were excluded from the study (Fig. 1).

Pathologic staging was carried out according to the current 7th edition of the TNM classification. Patients with T1N0M0, T2aN0M0, T2bN0M0, T3N0M0 and T4N0M0 tumors were identified as Stage IA, Stage IB, Stage IIA, Stage IIB and Stage IIIA respectively. The exclusion also applies to the stage IIIA patients who have direct tumor extension and are different from true node negative patients.

Information regarding the potential prognostic factors (gender, age, smoking status, tumor location, histology, tumor size, nLNs, extent of resection, adjuvant chemotherapy, pathological stage, differentiation and visceral pleural invasion) were identified under consideration. All of our patients were treated according to National Comprehensive Cancer Network (NCCN) guidelines.

### Ethics Statement

All patients gave written informed consent for their information to be stored in the hospital database and used for research. Ethical approval was obtained from Provincial Hospital Affiliated to Shandong University ethics committee.

### Follow-up

Follow-up information was ascertained from all patients through medical records or telephone interviews with the patient, a relative, or the referring physicians [9]. The evaluation involved the followings: chest X-ray, chest CT scan, abdominal ultrasonography, blood examination including pertinent tumor markers, and brain magnetic resonance imaging or bone scintigraphy if necessary.

We chose overall survival (OS) and disease free survival (DFS) as endpoints and investigated the associations between the potential prognosticators and these endpoints. OS was calculated, in months, from the date of the definitive resection to the time of death, censoring or last follow-up. DFS was calculated, in months, from the date of the definitive resection to the date of recurrence or distant metastasis, censoring or last follow-up.

### Statistical Analysis

Statistical analysis was performed using the SPSS 18.0 statistical software package. Descriptive statistics were used to describe the characteristics of the study cohort. Survival analysis and curves were established using the Kaplan-Meier method and log-rank test was used for comparison. Stepwise Cox proportional hazards model was used to estimate hazard ratios (HRs) and 95% confidence intervals (95% CIs) for each variable [10]. Multivariate analysis was performed to evaluate any possible association between nLNs and survival after adjusting for other potential confounders. As tumor size is a well-established independent prognostic factor [11], we particularly focused on the analyses stratified by nLNs combined with tumor size to compare survival among patients within the different groups. For nLNs and tumor size, the ‘optimal’ cutoff values were determined using *x^2^* scores, which were calculated by means of maximally selected log-rank statistics [10]. P values of less than 0.05 in a two-tailed test were considered to be statistically significant. Receiver operating characteristics (ROC) curve analysis using patients with pathologic stage I was performed to confirm whether the cutoff value of nLNs was equally applicable to all subsets of patients.

## Results

### Patient Characteristics

**Figure 2:**
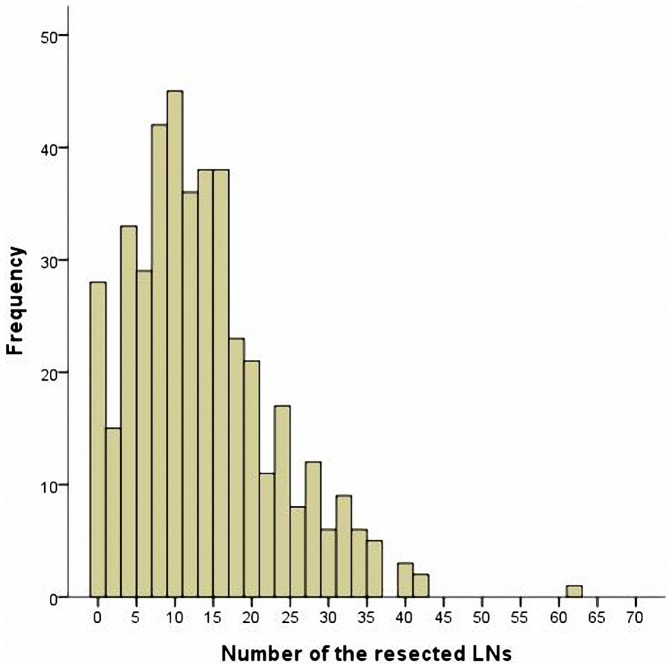
Distribution of the number of resected lymph nodes.

A retrospective series of 428 patients (293 men, 135 women; median age 60 years, range 23–84 years) who underwent surgery for NSCLC was identified from the original files of Department of Thoracic Surgery of Shandong Provincial Hospital. The patients’ characteristics were summarized in Table 1. The distribution of the number of lymph nodes in patients was shown in Fig. 2. Median number of resected nodes was 13 (range 0–61), with the median number of resected stations being 3 (range 0–7) (Table 2).

**Table 1:** Patients’ characteristics.

Variable	Category	No. of patients	%
Gender	Male	293	68.5
	Female	135	31.5
Age (years)	mean (range)	60.45(23–84)	
	≤65	253	59.1
	>65	175	40.9
Smoking status	Smoked	257	60.0
	Never smoked	171	40.0
Tumor location	Left	178	41.6
	Upper/lower	90/88	
	Right	250	58.4
	Upper/middle/lower	126/49/75	
Histology	Adenocarcinoma	234	54.7
	Squamous cell	158	36.9
	Other	36	8.4
Tumor size (cm)	mean (range)	3.55(0.3–11)	
	≤3.5	267	62.4
	>3.5	161	37.6
Number of resected LNs	mean (range)	13.18(0–61)	
Extent of resection	Wedge resection	29	6.8
	Segmentectomy	9	2.1
	Lobectomy	341	79.7
	Bilobectomy	34	7.9
	Pneumonectomy	15	3.5
Chemotherapy	Yes	164	38.3
	No	264	61.7
Pathological stage	I	335	78.3
	II	93	21.7
Visceral pleural invasion	Yes	157	36.7
	No	271	63.3

**Table 2:** Resected LNs’ characteristics.

Variable	Mean (range)	No. of patients	%
Total number of LNs resected	13.18(0–61)		
0		28	6.5
1–7		100	23.4
>7		300	70.1
N1 nodes resected	7.79(0–29)		
N2 nodes resected	5.36(0–33)		
Total resected LNs stations	2.65(0–7)		
N1 station	1.22(0–3)		
N2 station	1.44(0–5)		

### Cutoff Values for the Number of Lymph Nodes and Tumor Size

**Figure 3:**
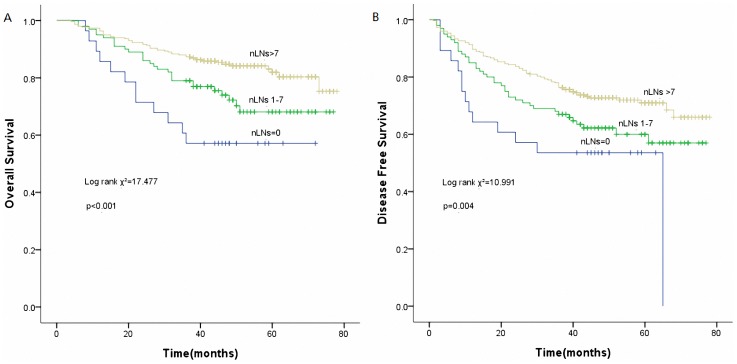
Survival curves of the number of resected lymph nodes (nLNs). (A) Overall survival curves of nLNs (*p*<0.001). (B) Disease free survival curves of nLNs (*p* = 0.004).

**Figure 4:**
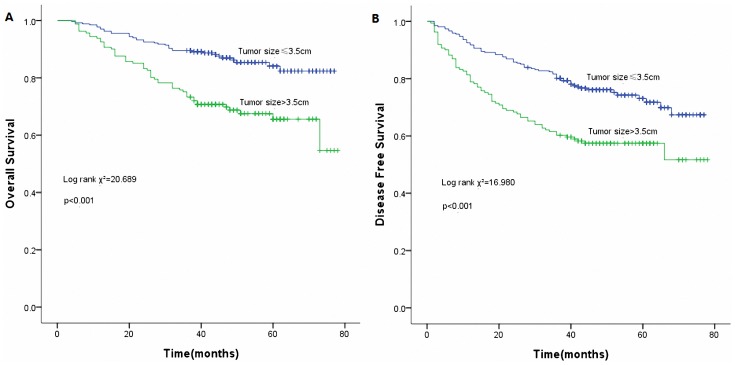
Survival curves of tumor size. (A) Overall survival curves of tumor size (*p*<0.001). (B) Disease free survival curves of tumor size (*p*<0.001).

Using the best cutoff approach by the maximally selected log-rank statistics [12], we identified 7 and 3.5cm as the optimal cutoff values for nLNs and tumor size respectively (Table 3). Unadjusted Kaplan-Meier curves stratifying patients according categorical nLNs and tumor size are shown in Fig. 3 and Fig. 4 respectively.

**Table 3:** Analysis of the number of resected lymph nodes and tumor size using the Cox proportional hazards model.

Cut-off value for lymphnode number	Chi-square score	*p*
0,1–5, >5	15.423	0.000
0,1–6, >6	16.485	0.000
0,1–7, >7	17.477	0.000
0,1–8, >8	14.183	0.001
0,1–9, >9	13.455	0.001
**Cut-off value for tumor size**	**Chi-square score**	***p***
≤2, >2	7.032	0.008
≤2.5, >2.5	11.781	0.001
≤3, >3	11.372	0.001
≤3.5, >3.5	20.689	0.000
≤4, >4	17.324	0.000
≤4.5, >4.5	15.132	0.000
≤5, >5	16.308	0.000

### Multivariate Analysis of Prognostic Factors

When all the prognostic factors were identified as categorical variables in the multivariate analysis (Table 4), nLNs, pathological stage and tumor size were independently prognostic for OS and DFS. Age and adjuvant chemotherapy had a significant impact on DFS instead of OS, while visceral pleural invasion showed a high discrimination power only for OS. In comparison with the baseline group (Nx), patients with 1 to 7 and >7 negative LNs had significantly better survival after adjusting for potential confounders (*p*<0.001 for OS and *p* = 0.001 for DFS). Besides, tumor size (*p* = 0.024), visceral pleural invasion (*p* = 0.006) and pathological stage (*p* = 0.012) were also associated with OS. Meanwhile, tumor size (*p* = 0.031), pathological stage (*p* = 0.030), age (*p* = 0.011) and chemotherapy (*p* = 0.029) were associated with DFS. Gender, smoking status, tumor location, histology and extent of resection were considerable factors in univariate analysis, but failed to attain predictive values in multivariate analysis. In addition, we found a statistically significant interaction between nLNs and tumor size (*p* = 0.001), indicating that the survival advantage among patients with a higher number of negative LNs may be limited to tumor size.

**Table 4:** Independent prognostic factor for OS and DFS by multivariate Cox regression analysis for the entire cohort of patients (n = 428).

Variable	OS		DFS	
	HR (95%CI) *p*		HR (95%CI) *p*	
nLNs		0.000		0.001
0	1		1	
1–7	0.489(0.245–0.977)		0.632(0.337–1.184)	
>7	0.206(0.106–0.403)		0.366(0.201–0.668)	
Pathological stage	2.000(1.165–3.435)	0.012	1.647(1.050–2.581)	0.030
Tumor size (cm)*	1.867(1.085–3.212)	0.024	1.587(1.042–2.417)	0.031
Visceral pleuralinvasion	1.841(1.189–2.851)	0.006	NS	
Age*	NS		1.568(1.110–2.214)	0.011
Chemotherapy	NS		1.483(1.041–2.112)	0.029

OS, overall survival; DFS, disease-free survival; HR, hazard ratio; 95% CI, 95% confidence interval; *p*, *p*-value; NS, not significant; nLNs, the number of resected lymph nodes;

* variables, considering 65 as age cutoff and 3.5cm as cutoff for nLNs.

### Survival and the Number of nLNs

Patients in our series were divided into three groups according to the total number of LNs: 0, 1–7, and more than 7(Fig. 3). Data from our study suggested that patients with more than 7 LNs removed had better OS (HR: 0.206, 95%CI: 0.106–0.403, p = 0.000) and DFS (HR: 0.366, 95%CI: 0.201–0.668, p = 0.001). In addition to other established prognostic factors, the number of resected LNs was an independent prognostic factor in both univariate and multivariate analysis.

**Figure 5:**
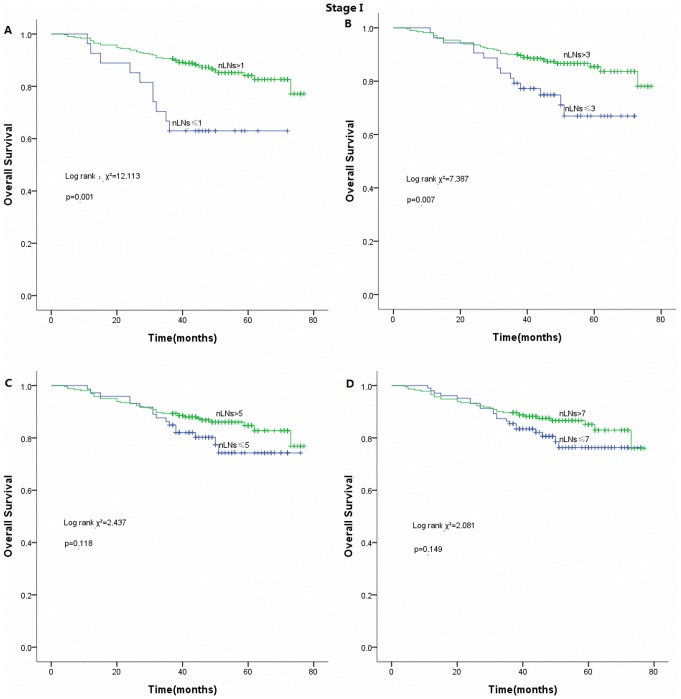
Survival curves of the number of resected lymph nodes (nLNs) in patients with stage I. (A) 1 resected LN as cutoff (*p* = 0.001). (B) 3 resected LNs as cutoff (*p* = 0.007). (C) 5 resected LNs as cutoff (*p>*0.05). (D) 7 resected LNs as cutoff (*p>*0.05).

**Figure 6:**
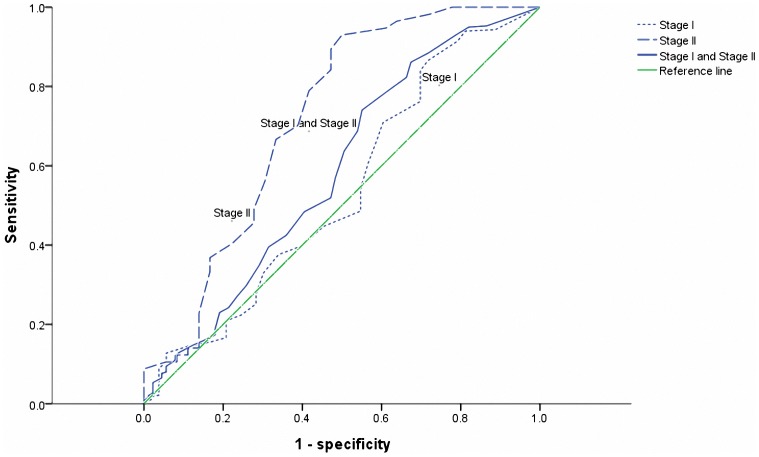
Receiver operating characteristics analysis based on the model of 7 resected lymph nodes in Stage I, Stage II and Stage I and Stage II results with OS as end point. In this model, the area under the curve (AUC) was 0.537, 0.586 and 0.719, respectively.

However, there was no statistically significant difference in survival (both OS and DFS) according to the model of 7 negative LNs in patients with pathological stage I (Fig. 5). The cutoff point with the highest sensitivity and specificity for estimating optimal negative LNs as a prognostic factor was set at 3 after ROC curve analysis (Fig. 6).

### Novel Predictor of Interest

**Figure 7:**
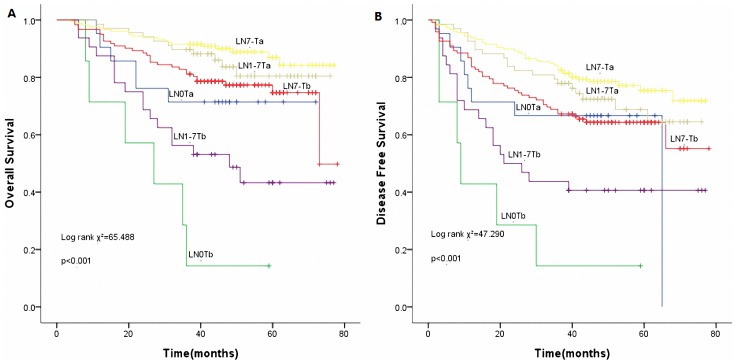
Survival curves of the new predictor (nLNs combined with tumor size). (A) Overall survival curves of the new predictor (*p*<0.001). (B) Disease free survival curves of the new predictor (*p*<0.001).

In order to be clear at a glance, tumor size less than or equal to 3.5cm was termed as ‘T_a_’ while tumor size more than 3.5cm was termed as ‘T_b_’. We classified the patients into 6 categories based on a combination of nLNs and tumor size as follows: LN_0_T_a_, LN_0_T_b_, LN_1–7_T_a_, LN_1–7_T_b_, LN_7-_T_a_ and LN_7-_T_b_. On multivariate analysis, the derived indicator was an independent prognostic factor for OS (*p*<0.001) as well as DFS (*p*<0.001). Fig. 7 has shown the survival curves of the new indicator. The distribution of the survival curves shows the survival benefit (both for OS and DFS) in proper order as follows: LN_7-_T_a_, LN_1–7_T_a_, LN_7-_T_b_, LN_0_T_a_, LN_1–7_T_b_, and LN_0_T_b_. Among them, LN_7-_T_a_ group had the most statistically significant OS (HR: 0.444, 95%CI: 0.251–0.785, *p* = 0.005) and DFS (HR: 0.550, 95%CI: 0.358–0.844, *p* = 0.006) benefit.

## Discussion

Here we report on the prognostic value of nLNs combined with tumor size in a series of surgically treated NSCLC patients. This information towards a better prognostication is worthy to help identify patients who would be candidates for more aggressive treatment or not [13], and stratify patients for clinical trials.

The NCCN guidelines recommend that ‘N1 and N2 node resection and mapping (ATS map)(minimum of three N2 stations sampled or complete lymph-node dissection)’ should be performed. Although serving consistently as a guide for therapy in clinical practice, pathologic nodal staging of lung cancer is often very poor. When remnant lung resection specimens were re-dissected after completion of pathology report, lymph nodes discarded were 1.5 times more than examined and 12% of pathologic node-negative patients had discarded metastatic lymph nodes [14]. These cases may introduce much uncertainty into pathologic nodal staging.

nLNs has been defined as a high risk factor in patients with lung cancer [10]. However, as current guidelines have not established an optimal surgical strategy with regard to nLNs. Its prognostic value and the minimum number of LNs to be examined are contentious issues. Several studies suggested that 11 to 16 was the optimal number of removed LNs to assess stage I lung cancer [5], [15]. Saji H et al. identified retrieval of 10 or more LNs may be warranted for evaluation of nodal status though there was no significant difference in survival for stage I patients [16]. They concluded that patients with 10 or more LNs had significantly worse outcomes than those with less than 10 LNs without considering nodal status. A recent report at ASCO Annual Meeting on the prognostic impact of nLNs in pN0 NSCLC demonstrated that examining 8 or more LNs improved survival [17]. Nevertheless, the variability of results maybe due to the heterogeneous populations conducted in the studies.

Our results suggested that without considering the stage, patients undergoing surgery for NSCLC should have at least 7 LNs removed. By further refinement, the model of 7 negative LNs was not statistically significant for the survival in patients with pathological stage I. However, the survival curves indicated that there still exists a better prognostic trend for patients with more than 7 LNs resected. The ROC curve analysis indicated that at least 3 LNs examined may be optimal for stage I NSCLC patients in our series. As less LNs in patients with small tumor size or early stage are tend to be examined, the therapeutic benefit is quite modest for this subset of patients. The alternative explanation is that it is less likely to harbor micrometastatic LNs. Furthermore, our work also showed that examining 7 negative LNs in stage II was more significant than in stage I, which suggested that the survival advantage in patients with a higher number of negative LNs may be limited to tumor size or visceral pleural invasion. It may be partly account for a possibility that tumors with large size and involved pleura rubbing contribute to cancer cell exfoliation and further lead to tumor recurrence and metastasis.

T stage and nLNs have been identified to stratify patients with pathologic N0 and Nx NSCLC [18]. Within our dataset, both nLNs and tumor size are demonstrated to be major independent prognostic factors for OS and DFS. Moreover, nLNs is closely associated with tumor size. The novel predictor (nLNs combined with tumor size) has a powerful discriminative ability regarding the prognosis of lung cancer (Fig. 7). Furthermore, as Fig. 7 shows, a clear tendency towards the improvement of OS and DFS from LN_0_ to LN_7-_ in the same tumor size category was observed. The removal of more than 7 LNs during surgery improved survival in lung cancer patients, particularly those with tumor size of less than 3.5cm. In this respect, nLNs in lung cancer has been proven to bear a prognostic potential, similar to that in other cancer types such as colon, breast, esophagus and gastric cancer [12], [13], [19], [20].

The most potential explanation for the association between nLNs and survival is stage migration. Patients categorized as node-negative may have had cancer disseminated to regional LNs. Hence, as the number of removed LNs increases during surgery, the probability of harboring micrometastatic LNs decreases and so does the proportion of stage migration, which is known as the Will Rogers phenomenon [21]. Additional immunohistochemical techniques to conventional ones may increase nLNs micrometastases found [7].

It is also worth mentioning that previous studies have shed light on the prognostic role of the number of positive LNs in lung cancer [9], [16], [22]. For patients in our institute, we have previously reported that more than 5 positive LNs removed and more advanced pN staging led to worse OS and DFS (*p*<0.0001) [9]. In Fukui et al.’s study, the survival curves showed significant stepwise deterioration as the number of positive LNs increased. The 5-year survival of patients with seven or more positive LNs was significantly the worst compared to those with 4–6, 1–3 or 0 positive LNs [22]. As the number of positive LNs has been extensively studied, it may provide additional information for the pN categories of the TNM classification. Wei S et al. suggested that staging by MLN were more accurate than the current pN stage which was based on anatomical location [23]. However, the benefits of the number of positive LNs were not available for treatment options during preoperative evaluation. Further insight into the role of the number of positive LNs in clinical application will depend on the prospects for development of imaging studies.

When interpreting the results of the current analysis, it is also important to consider the limitations of this study. First, it is a retrospective and single-institution analysis with a moderate sample size. Second, the definition of the optimal cutoff values of nLNs and tumor size needs to be further explored. Third, there is considerable practice variability among surgeons and pathologists, which may lead to discarded nodes in the operative specimen. Besides, nodal tissues may be divided into a few fragments or difficult to separate from the ‘en bloc’ dissected tissues. That is to say, it is possible that the true number of LNs examined may have been misestimated.

In conclusion, our study indicated that the total number of resected lymph nodes and tumor size are clinically important. It seems that nLNs combined with tumor size is an important independent predictor for survival in N0 and Nx patients with non-small cell lung cancer. We believe that the relatively simple, clinically-based, novel predictor may have a considerable impact on surgical resection. However, the discrimination power, potential mechanisms and performance for clinical practice should be validated in further large-scale cohort studies.
